# Phalangeal Intra-Articular Osteochondroma Caused a Rare Clinodactyly Deformity in Children: Case Series and Literature Review

**DOI:** 10.3389/fendo.2021.677245

**Published:** 2021-08-12

**Authors:** Yun Hao, Jia-Chao Guo, Xiao-Lin Wang, Jing-Fan Shao, Jie-Xiong Feng, Jin-Peng He

**Affiliations:** ^1^Department of Radiology, Tongji Hospital, Tongji Medical College, Huazhong University of Science and Technology, Wuhan, China; ^2^Department of Pediatric Surgery, Pediatric Orthopedic, Tongji Hospital, Tongji Medical College, Huazhong University of Science and Technology, Wuhan, China

**Keywords:** clinodactyly deformity, children, treatment, angulation, osteochondroma

## Abstract

**Background:**

Various factors are discovered in the development of clinodactyly. The purpose of this retrospective study was to present a group of children with a rare clinodactyly deformity caused by phalangeal intra-articular osteochondroma and evaluate the efficacy of various treatment methods.

**Methods:**

All child patients that were treated for finger problems in our center between Jan 2017 and Dec 2020 were reviewed. A detailed analysis was made of the diagnosis and treatment methods in eight rare cases. X-rays and histopathology were applied.

**Results:**

A preliminary analysis of 405 patients in total was performed, and we included eight cases in our final analysis. This cohort consisted of 2 girls and 6 boys, with a mean age of 5.74 ± 3.22 years (range: 2y5m to 11y). Overall, four patients had their right hand affected and four patients had their left hand affected. One patient was diagnosed as having hereditary multiple osteochondroma (HMO) while the other seven patients were all grouped into solitary osteochondroma. Osteochondroma was proven in all of them by histopathology examination. Preoperative X-rays were used to allow identification and surgery planning in all cases. All osteochondromas were intra-articular and in the distal end of the phalanges, which is located opposite the epiphyseal growth area. All of the osteochondromas developed in half side of the phalanges. The angulation in the finger long axis was measured, and resulted in a mean angulation of 34.63 ± 24.93 degree (range: 10.16-88.91 degree). All of them received surgery, resulting in good appearance and fingers straightening. No recurrence was recorded.

**Conclusions:**

This retrospective analysis indicates that 10 degrees can be selected as the angulation level for diagnosis of clinodactyly deformities. What’s more important, the abnormal mass proven by X-rays should be included as the classical direct sign for diagnosis. The first choice of treatment is surgery in symptomatic osteochondromas.

## Introduction

Clinodactyly is defined as a congenital curvature of a digit caused by an interphalangeal joint in the coronal plane (1). This curvature was generated by malalignment of the interphalangeal joint which was due to an abnormal trapezoidal or triangular shape of one or more phalanges (2–4). The visible curvature of the digit arises due to the abnormal shape that leads to asymmetric longitudinal growth in a direction from the original longitudinal axis of the finger. The fifth finger is the most frequently affected digit, but other fingers can be involved as well (5). Most cases are bilateral in this condition. However, the curvature angulation was reported quite differently in several pieces of research. Smith defined clinodactyly as an angulation deformity of the finger greater than 8° along the axis of phalanges (6). But some researchers argued that an angulation of less than 10° can also be normal, whereas others suggest that an angulation of greater than 15° should be abnormal (7). Samantha L. Piper reviewed thirteen digits in nine patients, and reported outcomes of opening wedge osteotomy to correct angular deformity in small finger clinodactyly, which showed all digits had greater than 20° of preoperative clinical angulation (mean 36°). But most of them agreed on curvatures with the coronal angulation of the affected digit greater than 10 degrees as a definition of clinodactyly.

Osteochondroma, one of the most common benign bone tumors, frequently occurs in the metaphysis of the long bones (8–10). According to the WHO’s data, it is detectable in 35% of benign bone tumors and 8% of all surgically removed bone tumors. Most clinodactyly deformities were developed by congenital deformity and delta phalanges, however, fewer cases were reported by osteochondromas (11–13).

The aim of treatment for clinodactyly is to improve aesthetics and function. Surgery is recommended if the deformity is severe and progressive. Clinodactyly affects the little finger in most cases. Most people can tolerate the deformity and work well without functional limitation. However, some specific activities can be hard for them to finish well, such as playing musical instruments, especially when the deformity is progressive. Physical therapy or surgery will be suggested for different patients with different conditions. A wedge osteotomy can correct the coronal deformity (5). In many reports, they can achieve good recovery and prognosis after receiving surgery, such as the opening wedge osteotomy. Complications are rarely seen, but we also noted the stiffness of the distal interphalangeal joint (14, 15). Here we reported a case series of rare clinodactyly deformity in children that was caused by solitary phalangeal intra-articular osteochondroma and provide our solution methods.

## Materials and Methods

A carefully review of 405 child patients who were treated for finger problems in our center (Tongji Hospital, Tongji Medical College, Huazhong University of Science and Technology) between Jan 2017 and Dec 2020 was finished. Overall, 63.9% children cases underwent surgery for finger deformity, 24.5% of them underwent surgery for trauma, 6.4% of them underwent surgery for tumor, 5.2% of them underwent surgery for infection, while only eight child patients underwent surgery for phalangeal intra-articular osteochondroma. Therefore, only eight pediatric patients were brought into this retrospective study. Eight cases were reviewed for diagnosis of pediatric phalangeal intra-articular osteochondroma that caused a rare clinodactyly deformity.

This case series included 2 girls and 6 boys, ranked with a mean age of 5.74 ± 3.22 years (range: 2y5m to 11y). Overall, four cases had the left arm affected and four cases had the right arm affected. Patient demographics and details of the surgery treatment methods were obtained from the electronic medical records system (**Table 1**). One patient was diagnosed as having hereditary multiple osteochondroma (HMO) while the other seven patients were all grouped into solitary osteochondroma. Preoperative X-ray scans were done to make identification and surgery planning before operation for all children. We can evaluate the accurate angulation along the finger long axis between the proximal and middle phalanges using pre-operation X-rays. All fingers with angular deformities were requested for surgery. Hereditary multiple exostosis is a bony dysplasia in which osteochondromas affect multiple long and flat bones. Just like osteochondromas, they are usually defined as masses occurring in the metaphyseal region and adjacent to the growth plate. However, unlike those traditional osteochondromas, we reported those eight special cases of intra-articular osteochondromas located at the distal end of the phalanges opposite the epiphyseal growth area. The fixation method for all cases but one fingers was a single 0.039-inch Kirschner wire (K-wire). Those eight patients were fixed for 4 to 6 weeks (generally 5 weeks), after which the K-wire was removed to allow rehabilitation training. The study obtained ethical approval from the Review Board of Tongji Hospital ethical committee, and all patients gave written informed consent. This was a retrospective case-control study.

**Table 1:** Clinical materials and treatment methods for 8 patients.

No.	Sex	Age	Side	Finger	Joint	Phalange	Orientation	Mass location	Angle	Range of motion	K-wire fixation	Appearance	Recurrence	Joint stiffness
1	Boy	8y1m	Right	Second	DIP	Middle	Radial	Ulnar half side	42.55	Severe limitation	Yes	Straight	No	No
2	Boy	3y3m	Right	Second	PIP	Proximal	Ulnar	Radial half side	25.96	Limitation of externsion	Yes	Straight	No	No
3	Boy	9y1m	Right	Forth	PIP	Proximal	Ulnar	Radial half side	15.43	No limitation	Yes	Straight	No	No
4	Boy	2y10m	Left	Third	PIP	Proximal	Radial	Ulnar half side	41.79	No limitation	Yes	Straight	No	No
5	Girl	2y5m	Left	Forth	PIP	Proximal	Radial	Ulnar half side	88.91	Limitation of externsion	Yes	Straight	No	No
6	Girl	4y6m	Left	Third	PIP	Proximal	Radial	Ulnar half side	10.16	No limitation	No	Straight	No	No
7	Boy	4y9m	Right	Forth	PIP	Proximal	Ulnar	Radial half side	19.01	No limitation	Yes	Straight	No	No
8	Boy	11y	Left	Forth	PIP	Proximal	Ulnar	Radial half side	33.23	Limitation of externsion	Yes	Straight	No	No

## Results

The fourth finger was the most frequently affected finger with four patients, followed by the third and second fingers with two patients each. The proximal phalanx was the most frequently involved (seven patients), and the osteochondroma affected the proximal interphalangeal joint (PIP) in seven cases. Only in one patient was the middle phalanx affected and the distal interphalangeal joint (DIP) was involved. All osteochondromas were intra-articular and in the distal end of the phalanges opposite the epiphyseal growth area. All of the osteochondromas developed in half side of the phalanges. Four patients developed in the ulnar side and the other four patients developed in the radial side. Thus, four fingers oriented to the radial side and four of them oriented to the ulnar side. The angulation in the finger long axis was measured, resulting in a mean angulation of 34.63 ± 24.93 degree (range: 10.16-88.91 degree). All of them received operation, which resulted in good appearance and fingers straightening. No recurrence was recorded.

All of them were proven to be osteochondroma by histopathology examination. According to what was discovered in the surgery, we applied different operations for each patient. One patient received a surgery without K-wire fixation since the finger was automatically straightened after mass resection. The other seven patients were all fixated by K-wire to stretch the finger. The joint was opened in all patients while ligament reconstructions were not carried out.

### Case 1 Mass Resection With K-Wire Immobilization in a Solitary Osteochondroma

**Figure 1:**
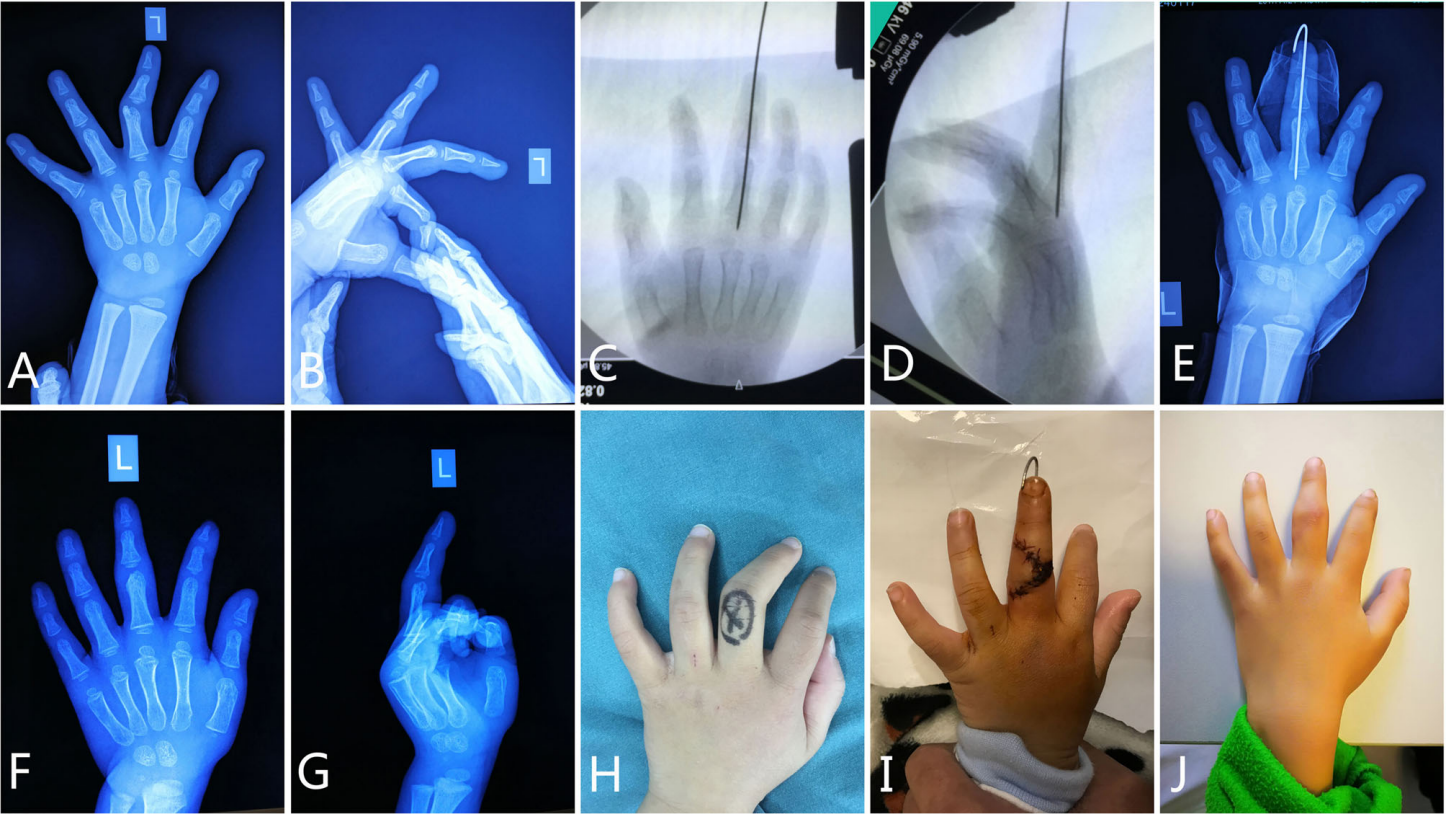
Diagnosis and treatment of clinodactyly deformity in a 2-year-10-month-old boy. The radiograph on presentation showed an outgrowth around the distal end of the third proximal phalanx **(A, B)**. During the surgery, the abnormal mass was resected and a K-wire was implanted to keep the finger straight **(C, D)**. After operation, the radiographs showed good prognosis **(E–G)**. Clinical examination established the diagnosis of clinodactyly deformity of his third finger **(H)**, and also showed essentially normal movement of the upper extremity at the follow-up **(I, J)**.

**Figure 2:**
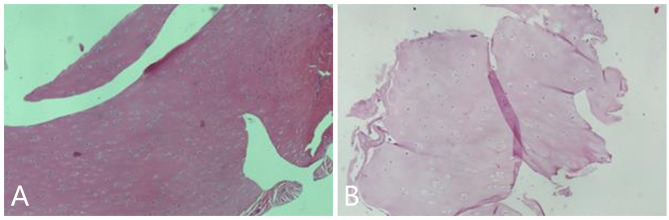
The pathology examination of the resected mass showed cartilage map **(A, B)**.

A 2-year-10-month old boy presented to the pediatric orthopedic surgeon with complaints of clinodactyly deformity in his third left finger with a duration of over 2 years. There was no complaint of pain and extension motion. He had no history of symptoms of infectious diseases such as fever or significant trauma. A hard mass could be also felt near the proximal interphalangeal joint. No tenderness or pain was present, and there was also no restricted movement range. Whether for the plantar flexion or dorsiflexion, his finger’s function is normal comparing with the right hand. We had no evidence of any signs of inflammation to relate to secondary deformity when analyzing the normal skin. There was no familiar history. The patient was sent to the radiologist for X-ray evaluation (**Figures 1A, B**). Anteroposterior radiographs of his hands showed of an osseous outgrowth mass from the ulnar aspect of the distal end of the proximal phalange opposite the epiphysis plate (**Figures 1A, B**). The clinical manifestation of the finger and characteristic radiographic images established the diagnosis of clinodactyly deformity. Because the patient had presented with swvere deformity, surgical treatment was considered (**Figures 1C–E**). An immediate and significant improvement was seen on X-ray, demonstrated postoperatively (**Figures 1F, G**). The physical examination showed a bending finger which twisted toward the radial side (**Figure 1H**). A dorsal approach was preferred and the proximal interphalangeal joint capsule was opened (**Figure 1I**). The abnormal outgrowth mass was excised, and an attempt to maintain finger straightening by immobilization of a K-wire was made. An immediate and significant improvement was seen on appearance, demonstrated perioperatively and postoperatively (**Figure 1J**). The pathology showed osteochondroma (**Figures 2A, B**).

### Case 2 Mass Resection Without K-Wire Immobilization in a Solitary Osteochondroma

**Figure 3:**
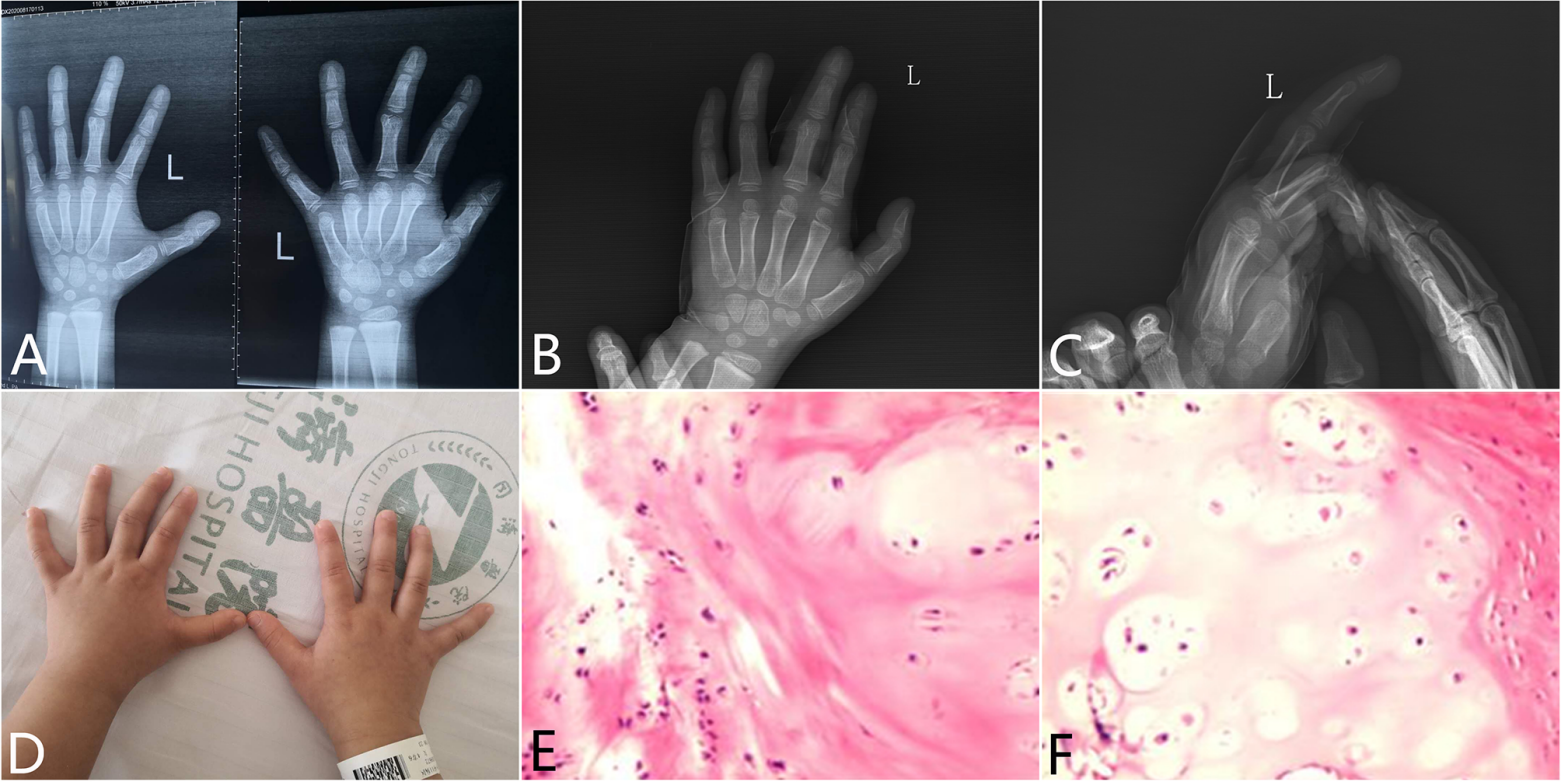
Diagnosis and treatment of clinodactyly deformity in a 4-year-6-month-old girl. X-ray radiography showed a mass protruded into the PIP joint which made the axis declined **(A)**. During the surgery, the abnormal mass was resected without K-wire implantation **(B, C)**. Clinical examination established the diagnosis of clinodactyly deformity of her third finger **(D)**. Pathology examination proved to be osteochondroma **(E, F)**.

Another patient was female, aged 4 years and 6 months. No pain at the joint was present. Function was normal. The patient had no other history of diseases associated with musculoskeletal or neurological abnormal problems. An accessory growth protrusion in the distal end of the proximal third phalanx was proven by radiographys (**Figure 3A**). The lesion was located on the ulnar side of the phalanx and was resected through a surgery. K-wire was not applied in this surgery (**Figures 3B, C**). At 4 years of age, the mass was discovered on the third finger of her left hand and an axis line deflection was denoted (**Figure 3D**). After surgery, a histopathology confirmed the osteochondroma (**Figures 3E, F**). Her finger points with preservation of a correct axis with full mobility. Follow-up revealed a good prognosis.

### Case 3 Mass Resection With K-Wire Immobilization in a HMO

**Figure 4:**
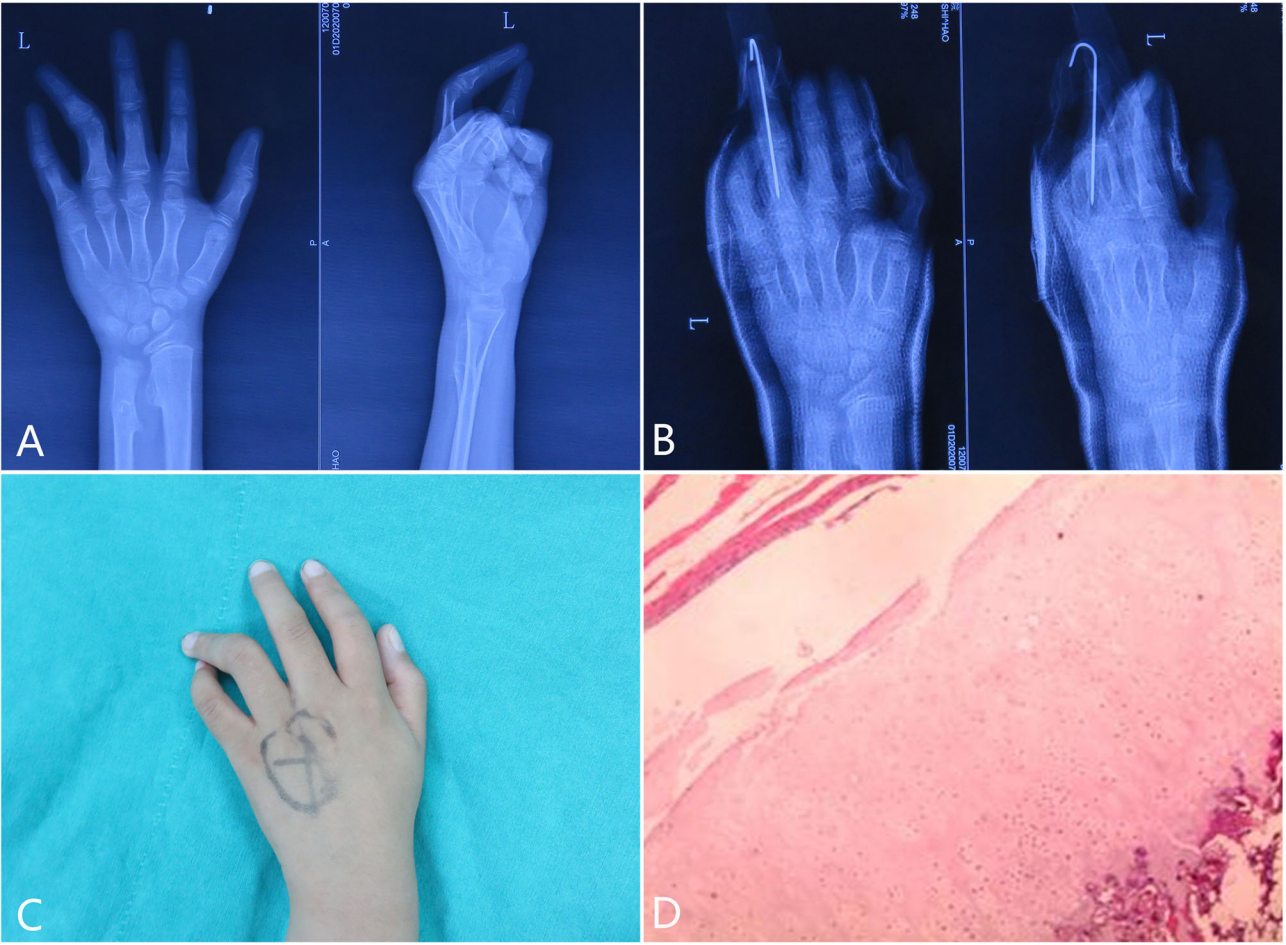
Diagnosis and treatment of clinodactyly deformity in an 11-year-old boy. The radiograph on presentation showed an outgrowth around the distal end of the fourth proximal phalanx **(A)**. During the surgery, the abnormal mass was resected and a K-wire was implanted to keep the finger straight **(B)**. Clinical examination established the diagnosis of clinodactyly deformity of his third finger **(C)**. Pathology examination proved to be osteochondroma **(D)**.

An 11-year-old boy presented to our hospital with progressive bowing of his left fourth finger. He had no history of trauma. We detected no deficit in strength for his finger. On physical examination, there was a palpable mass located intra-articular. Plain radiography of left hand showed a mass-like tumor arising from the dotsalradio portion of the head of the fourth proximal phalanx (**Figure 4A**). We also found several osteochondromas located at the metaphysis of radius and ulnar bones (**Figure 4A**). What’s more, multiple osteochondromas were discovered around his limbs. Intraarticular osteochondroma located in the intraarticular portion of proximal phalanx was identified during surgery. The tumor was completely excised, and a K-wire was implanted to make the finger straight (**Figure 4B**). On physical examination, extension was limited to 30° compared to the other side (**Figure 4C**). A general histopathology confirmed the diagnosis of osteochondroma after surgery (**Figure 4D**). The lesion had a cartilage cap without signs of necrosis. No atypia or binucleate chondrocytes were found under the microscope by histologic examination. After surgery, the patient had an uneventful recovery with normal function of the joint.

## Discussion

Clinodactyly is a common congenital hand abnormality defined as finger deviation in the coronal plane; it affects the fifth finger most frequently. Clinodactyly occurs in approximately 1% of normal newborns, but incidence of clinodactyly has been reported to be as high as 5% in the Japanese population (16). Some clinodactyly cases were classified as brachydactyly for its shortening change of the involved finger (17). There are many clinical features characteristic of abnormal phalanx; the most common are large, rounded phalangeal blocks as reported in Cenanie-Lenz syndrome, delta phalanges combined with a C-shaped epiphysis, and trapezoidal phalanges with a double epiphysis (1, 18–21). It is difficult to make an early differential diagnosis between these abnormal phalanges, but it is important for those patients to receive appropriate treatment. Clinodactyly in many pediatric patients is a problem of unbalanced longitudinal growth from a bracketed epiphysis. There is huge debate over which type of surgical intervention to choose and when to operate. It is a little different from our cases which were caused by phalangeal intra-articular osteochondroma prominence. Appearance but not functional impairment is the major problem that we often meet. Many studies approved an angular deviation of fewer than 10 degrees as normal variant. Therefore, clinodactyly is defined as the coronal deviation of the affected digit greater than 10 degrees. We should avoid early inappropriate surgery before correct diagnosis. However, surgery was suggested in those cases with deviation over 20 degrees and associated with shortening of the phalange (22). Clinodactyly is mistaken for many finger diseases that cause a curved finger. Clinodactyly is also found in a large group of congenital abnormalities and presented as one single sign. Therefore, it is a special group of clinical sign, and this curvature may be found at birth, may develop gradually, or may be secondary to a trauma or infection diseases. If the phalangeal epiphyses were destroyed by trauma or inflammation, the growth may be disturbed and may lead to angulation deformity. This growth disorder may also be caused by congenital reasons such as an abnormally shaped phalanx (23).

Osteochondroma, one of the most common benign bone tumors, frequently occurs in the metaphysis of the long bones (8). Osteochondroma is called a benign cartilage-forming tumor and arises from an aberrant subperiosteal cartilage. Multiple osteochondromas syndrome (MOS) is an autosomal dominant disease which has mutations in the EXT (EXT1 or EXT2) genes (9, 10). Osteochondroma is one of the most common benign bone tumors. According to the WHO data, it is detectable in 35% of benign bone tumors and 8% of all surgically removed bone tumors (24). Most clinodactyly deformities were developed by congenital deformity and delta phalanges, however, fewer cases were reported by osteochondromas (11–13, 25). In Goo Hyun Baek’s report (13), only 7 of 10 patients were children aged below 12 years, and four patients presented without coronal deformity. Therefore, it is different from our cohort who all have evident coronary angulation over 10 degrees. Here we reported a special kind of clinodactyly deformity that is caused by intra-articular osteochondromas, including solitary intra-articular osteochondroma and hereditary multiple osteochondroma.

Through our case series, it was seen that boys developed this disease more frequently than girls. And this disease mainly affected the fourth finger, but can also develop on the second and third finger. The main complaint was bending of the finger. In this rare kind of clinodactyly deformity, angulation in the long finger axis between the two different phalanges ranked from 10.16 degree to 88.91 degrees, according to X-rays. Thus, we suggested 10 degrees can be selected as the angulation level of such a group of clinodactyly deformity. What’s more important, the abnormal mass proven by X-rays should be included as the classical direct sign for diagnosis. We can easily find out that the intra-articular mass protruded into the joint lead to angulation of the finger, which will worsen as the mass grows. Histopathology examination proved this mass to be diagnosed as intra-articular osteochondroma. We have found a cartilage cap with enchondral bone formation beneath the cartilaginous cap through HE staining. Dysplasia epiphysealis hemimelica (DEH), or Trevor’s disease, is a relatively rare disorder for asymmetric epiphyseal cartilage overgrowth or an accessory epiphyseal ossification center (11). But it was initially reported as a kind of foot disorder (tarsomegalia). However, we are unable to diagnosis this special disease as Trevor’s disease since there is no epiphysis at the distal end of phalanges.

In Goo Hyun Baek’s report (13), two patients had been neglected for a long time. These two patients had relatively poor prognosis due to the development of finger osteoarthritis, residual deformity, and permanent limitation of finger motion. Thus we should be aware of neglected intra-articular phalangeal osteochondroma which will cause progressive deformity and limitation of motion. Although Vickers’s physiolysis and a variety of wedge osteotomy are both common treatment options for clinodactyly, there are various forms of complications including infection, joint stiffness, nonunion of osteotomy, and disease recurrence (26). In our case series, the osseous mass was excised in seven cases, including six cases of solitary intra-articular osteochondroma and one case of hereditary multiple osteochondroma. Temporary fixation of the distal interphalangeal joint by a Kirschner wire was used in these patients. However, we also only resected this mass in one case of solitary intra-articular osteochondroma without Kirschner wire fixation since the clinodactyly reduced spontaneously. No ligamentary adjustment seemed necessary since the treatment was early and there was little destruction of those ligaments. Kirschner wire fixation was applied to correct the clinodactyly. Temporary fixation will not cause joint stiffness. However, ligamentary adjustment may result in joint motion limitation. But the surgeons should carefully make the surgery plan and accurately define the resection margin. The follow-up was uneventful. No recurrence was found in follow-up. All fingers developed well in a normal axis combined with full ranges of motion. Our findings suggest an early surgical approach is appropriate in these definite cases.

In summary, phalangeal intra-articular osteochondroma caused a rare clinodactyly deformity in children in our report. Only several cases were discovered. The first choice of treatment is surgery. Skeletal deformity will progress throughout childhood, which will cause finger malfunction and even joint degeneration. However, a longtime follow up is still absent for this surgery. We should pay specific attention to the destruction of some articular cartilage when mass is removed. Thus we need to distinguish the osteochondroma cartilage from normal articular cartilage carefully. Furthermore, the limitations of the present study include its retrospective nature, a relatively small study group, limited follow-up in some cases, and the lack of a control group.

## Data Availability Statement

The original contributions presented in the study are included in the article/Supplementary Material. Further inquiries can be directed to the corresponding author.

## Ethics Statement

The studies involving human participants were reviewed and approved by the Review Board of Tongji Hospital ethical committee. Written informed consent to participate in this study was provided by the participants’ legal guardian/next of kin. Written informed consent was obtained from the minor(s)’ legal guardian/next of kin for the publication of any potentially identifiable images or data included in this article.

## Author Contributions

J-PH and YH performed the research and analyzed the data. X-LW, C-JG, J-FS and J-XF analyzed the data. J-PH and J-FS designed the study. YH, J-PH and J-FS supervised the study. YH and J-PH wrote the paper. All authors contributed to the article and approved the submitted version.

## Funding

This work was supported by the Primary Research & Developement Plan of Hubei Province (Grant No. 2020BCB006) and the Science and Technology Innovation Base Platform (Grant No. CXPTZX003).

## Conflict of Interest

The authors declare that the research was conducted in the absence of any commercial or financial relationships that could be construed as a potential conflict of interest.

## Publisher’s Note

All claims expressed in this article are solely those of the authors and do not necessarily represent those of their affiliated organizations, or those of the publisher, the editors and the reviewers. Any product that may be evaluated in this article, or claim that may be made by its manufacturer, is not guaranteed or endorsed by the publisher.
